# Anaplastic thyroid carcinoma: three protocols combining doxorubicin, hyperfractionated radiotherapy and surgery

**DOI:** 10.1038/sj.bjc.6600361

**Published:** 2002-06-17

**Authors:** J Tennvall, G Lundell, P Wahlberg, A Bergenfelz, L Grimelius, M Åkerman, A-L Hjelm Skog, G Wallin

**Affiliations:** Department of Oncology, Lund University Hospital, SE-221 85 Lund, Sweden; Department of Otorhinolaryngology, Lund University Hospital, SE-221 85 Lund, Sweden; Department of Surgery, Lund University Hospital, SE-221 85 Lund, Sweden; Department of Pathology/Cytology, Lund University Hospital, SE-221 85 Lund, Sweden; Department of Radiumhemmet, Karolinska Hospital, SE-104 01 Stockholm, Sweden; Department of Surgery, Karolinska Hospital, SE-104 01 Stockholm, Sweden; Department of Pathology/Cytology, Karolinska Hospital, SE-104 01 Stockholm, Sweden; Department of Genetics and Pathology, Uppsala University, SE-751 85 Uppsala, Sweden

**Keywords:** anaplastic thyroid carcinoma, combined treatment modality, doxorubicin, accelerated radiotherapy, surgery

## Abstract

Patients with anaplastic thyroid carcinoma can rarely be cured, but every effort should be made to prevent death due to suffocation. Between 1984 and 1999, 55 consecutive patients with anaplastic thyroid carcinoma were prospectively treated according to a combined regimen consisting of hyperfractionated radiotherapy, doxorubicin, and when feasible surgery. Radiotherapy was carried out for 5 days a week. The daily fraction until 1988 was 1.0 Gy×2 (A) and 1989–92 1.3 Gy×2 (B) . Thereafter 1.6 Gy×2 (C) was administered. Radiotherapy was administered to a total target dose of 46 Gy; of which 30 Gy was administered preoperatively in the first two protocols (A and B), while the whole dose was given preoperatively in the third protocol (C). The therapy was otherwise identical. Twenty mg doxorubicin was administered intravenously weekly. Surgery was possible in 40 patients. No patient failed to complete the protocol due to toxicity. In only 13 cases (24%) was death attributed to local failure. Five patients (9%) ‘had a survival’ exceeding 2 years. No signs of local recurrence were seen in 33 patients (60%); 5 out of 16 patients in Protocol A, 11 out of 17 patients in Protocol B, 17 out of 22 patients in Protocol C (*P*=0.017). In the 40 patients undergoing additional surgery, no signs of local recurrence were seen in 5 out of 9 patients, 11 out of 14 patients and 17 out of 17 patients, respectively (*P*=0.005).

*British Journal of Cancer* (2002) **86**, 1848–1853. doi:10.1038/sj.bjc.6600361 www.bjcancer.com

© 2002 Cancer Research UK

## 

Anaplastic thyroid carcinomas (ATC), in sharp contrast to differentiated thyroid carcinomas, have a poor prognosis (Aldinger *et al*, 1978; Tan *et al*, 1995; Ain, 1998). Most patients suffering from ATC die due to uncontrolled local tumour invasion causing suffocation (Jereb *et al*, 1975; Junor *et al*, 1992). ATC is a rare disease and the vast majority of the patients affected are older than 60 years (Nel *et al*, 1985; Demeter *et al*, 1991; Tan *et al*, 1995; Ain, 1998). Treatment must consequently be governed by conditions associated with high age. Although patients with ATC can rarely be cured, every effort should be made to control the primary tumour and thereby improve the quality of the remaining life.

Surgery, radiotherapy, or chemotherapy used alone is seldom sufficient to control the disease (Aldinger *et al*, 1978; Venkatesh *et al*, 1990), while a combination of these modalities may improve local control (Venkatesh *et al*, 1990; Schlumberger *et al*, 1991). The rationale for combining radio- and chemotherapy is that, as the toxicity of these modalities does not entirely overlap, an enhanced tumouricidal effect may be obtained (Wendt *et al*, 1985).

The most effective and most commonly used single cytostatic agent against thyroid carcinomas is doxorubicin (Ekman *et al*, 1990). The combination of doxorubicin and radiation in mammalian tumour cells is synergistic when a low dose of this cytostatic agent (<0.15 mg kg^−1^) is used (Byfield *et al*, 1977). The mechanism responsible for the radiosensitising effect of doxorubicin is a subject of speculation (Rosenthal and Rotman, 1991).

Hyperfractionated radiotherapy can reduce the early reaction in normal tissues (Withers, 1985; Rosenthal and Rotman, 1991). As ATC is a rapidly growing tumour, it may be important to decrease the ‘overall treatment time’ by accelerating the fractionation of the radiotherapy regimen, thereby reducing the opportunity for tumour cells to repopulate during the course of treatment (Thames *et al*, 1982). Moreover, surgery can remove the large necrotic tumour mass, which theoretically may enhance the efficacy of the other treatment modalities (Schlumberger *et al*, 1991).

Based on the above rationale, we have prospectively evaluated a combined regimen consisting of hyperfractionated radiotherapy, doxorubicin, and, when feasible, debulking surgery in 55 consecutive patients presenting with ATC. The hyperfractionated radiotherapy was gradually accelerated in each of the three protocols evaluated, while the total radiation dose remained the same. The main purpose of this present study, which is to our knowledge the largest prospective study of ATC, was to improve the local control rate, a prerequisite for cure and decent quality of life.

## PATIENTS

Between August 1984 and January 1999, 67 consecutive patients with ATC, cytologically verified by fine-needle aspiration, were referred to the Departments of Oncology in Lund (*n*=35) or in Stockholm, Radiumhemmet, (*n*=32). Fifty-five patients entered one of the three sequential protocols. The reasons for excluding 10 patients were: refusal to accept treatment (*n*=1), lack of co-operation due to senility (*n*=2); major protocol violations (*n*=4); and a discrepancy between the diagnosis based on aspiration cytology and subsequent histopathologic findings at surgery (*n*=3). In these three cases the histologic diagnosis was a poorly differentiated thyroid carcinoma, a poorly differentiated medullary thyroid carcinoma, and a poorly differentiated metastasis. This discrepancy in diagnosis may be due to therapeutic eradication of microscopic foci of anaplastic carcinoma in the poorly differentiated thyroid carcinoma. This diagnostic approach meant that a few patients with poorly differentiated carcinomas received preoperative chemo- and radiotherapy. The alternative approach of a diagnostic surgical biopsy would have resulted in a delay in the initiation of therapy because of poor healing of the surgical scar.

At the time of presentation each of the 55 patients had a rapidly enlarging neck mass. In 13 patients, a nodular goitre had been previously recognised. Due to the enlarging thyroid mass, patients had symptoms from compression or invasion of the upper aerodigestive tract such as dyspnea (*n*=16), dysphagia (*n*=13), hoarseness (*n*=11), and local pain (*n*=7). The median duration of symptoms prior to diagnosis was 1.5 months (range, 0–8 months).

Table 1A

**Table 1:**
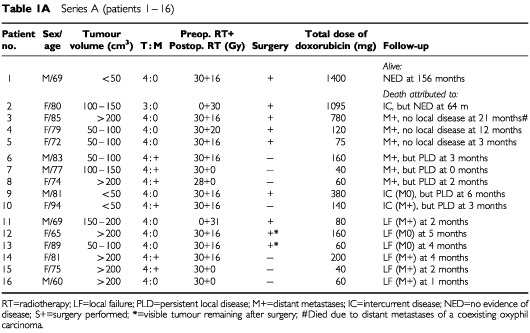
Series A (patients 1 – 16)

[Table tbl1b]

**Table tbl1b:**
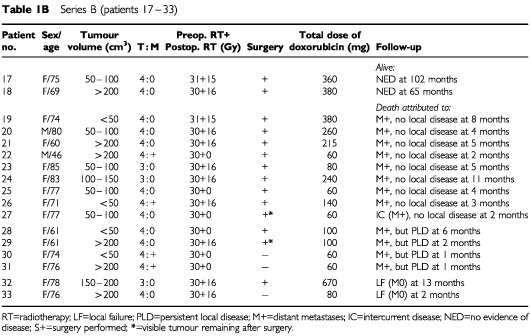
Series B (patients 17 – 33)

–1c

**Table tbl1c:**
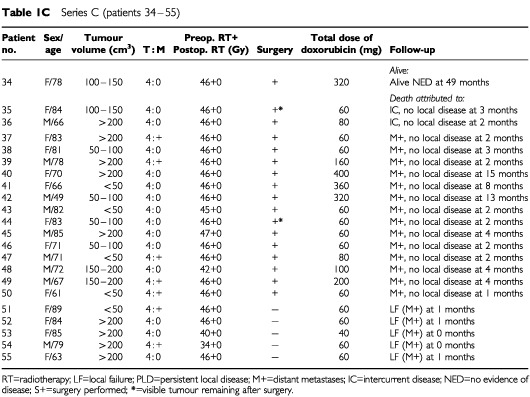
Series C (patients 34 – 55)

presents the patient characteristics and the tumour extension at the start of therapy. Ages ranged between 46 and 94 years (median 76) with a preponderance of females (*n*=38 *vs* *n*=17). The large thyroid tumours often extended into surrounding tissues (T4; *n*=51), making preoperative evaluation of regional lymph nodes difficult. Eighteen of the patients with T4 tumours had vocal cord paralysis. The remaining four patients (Patients 2, 23, 24 and 32 in Table 1) had large but intrathyroidal tumours (T3).

Tumour volume was estimated prior to the start of treatment according to the formula for a rotating ellipsoid (volume=length×width^2^/2), which gives a good approximation of tumour weight. The estimated tumour volume was less than 50 ml in 12 patients, 50–200 ml in 22 patients, and exceeded 200 ml in 21 patients.

At diagnosis, the disease was limited to the neck in 38 patients, while pulmonary metastases were diagnosed in the remaining 17 patients. In our experience, surgical biopsy delays the initiation of therapy due to poor healing of the surgical scar (Nilsson *et al*, 1998), and as cytology is reliable in diagnosing ATC (Löwhagen *et al*, 1981; Åkerman *et al*, 1985), the diagnosis in 51 of the 55 patients was established by fine-needle aspiration. On entering the study, only two patients had received any previous anti-tumour therapy. In these two patients (Patients 2 and 11), the initial treatment was surgery and subsequent histopathology provided the diagnosis. All patients were reevaluated with regard to the diagnosis including a review of all histopathologic specimens. In a few doubtful cases, additional immunocytochemistry confirmed the epithelial origin of the tumours. Classification was made according to the World Health Organisation (WHO) criteria. If the patients did undergo surgery, the diagnosis was based on the results of the aspiration cytology and the clinical data.

## METHODS

**Figure 1:**
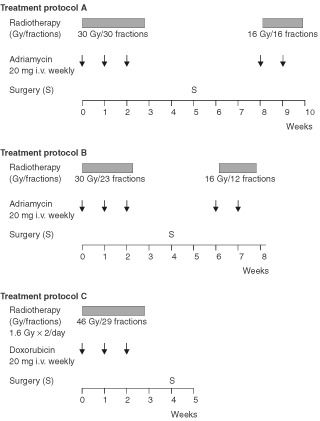
Treatment protocol for the three consecutive series A, B and C.

After the diagnosis of ATC had been established cytological, therapy was started immediately with informed consent being given by all 55 patients. The treatment regimens are presented in Figure 1

: A, patients 1–16 treated in 1984–1988, B, patients 17–33, treated in 1989–1992 and C, patients 34–55, treated in 1993–1999. Preliminary data for Protocols A and B have been presented earlier (Tennvall *et al*, 1994). The combined treatment consisted of hyperfractionated radiotherapy, doxorubicin, and when feasible, surgery. A dose of 20 mg doxorubicin was administered once weekly prior to the first radiotherapy session.

In Protocol A radiotherapy was administered preoperatively to a target dose of 30 Gy during 3 weeks and an additional 16 Gy postoperatively during 1.5 weeks, resulting in a total target dose of 46 Gy within 70 days. It was administered twice daily, 5 days a week, with a target dose of 1 Gy per fraction and with a minimum interval of 6 h.

In Protocol B hyperfractionated radioptherapy was accelerated by administering a target dose of 1.3 Gy per fraction twice daily to the same total doses pre- and postoperatively. The overall treatment time for the local therapy was consequently shortened to approximately 50 days but radiotherapy was otherwise the same.

In Protocol C, the radiotherapy was further accelerated by increasing the target dose to 1.6 Gy/fraction twice daily and administering all the radiation preoperatively, *viz* 46 Gy in 29 fractions (1.6 Gy×2 per day) within 3 weeks. Since the radiotherapy was accelerated, the minimum interval of 6 h between the two fractions was even more important so as to prevent spinal cord myelopathy (Dische and Saunders, 1989; Wong *et al*, 1991).

The patients were treated in the supine position. Two opposed fields (AP–PA) were used, encompassing, in addition to the thyroid tumour (target 1), the regional lymph nodes of the neck, the supraclavicular areas, and the upper mediastinum (target 2). Spinal cord shielding was used in the posterior portal during postoperative radiotherapy. The radiotherapy was delivered with a high-voltage technique (60 Co, 4 MV, or 6 MV photons). If the tumour had infiltrated superficial tissues, a bolus was used. The dose to target 1 was estimated to vary from 46 to 50 Gy, except behind the spinal cord shield, where the dose was 3–4 Gy lower (*viz* a maximum dose of 46 Gy in all three protocol). This implied a total variation of the specification dose within target 1 ranging from −8% to +10%.

The aim of thyroid surgery was to remove all visible tumour. If the planned operation was abandoned in Protocols A and B, the second course of radiotherapy was started within 3 weeks. In Protocol C all planned radiotherapy was administered in one series preoperatively and further radiotherapy was in no case considered. The two patients (numbers 2 and 11) who had undergone prior surgery in Protocol A received only one course of postoperative hyperfractionated radiotherapy to a dose of 30 Gy, in combination with doxorubicin (Table 1).

After the completion of the local treatment, the weekly administration of doxorubicin at 20 mg i.v. was continued in patients showing sufficiently good performance (Table 1). The upper scheduled level of doxorubicin was 750 mg m^2^ in Protocol A. The schedule for Protocols B and C was doxorubicin therapy for a maximum of 3 months after termination of the local therapy corresponding to a total dose of 430–450 mg. Statistical comparisons were performed with Fisher's exact test (two-sided) and the χ^2^ test for trend (Altman, 1991).

## RESULTS

### Feasibility

All three protocols could be carried out. Local toxicity: the forms of acute toxicity observed were mucosal and skin reactions corresponding to WHO grades 1 or 2 apart from four patients; one each in Protocols A and B and 2 in Protocol C. Two of these patients (belonging to Protocol A and B) required a break of 1–1.5 weeks before resumption of therapy and one patient required an enteral tube after termination of the therapy. Thus, all but these four patients with oesophagitis WHO grade 3 (*n*=3) or grade 4 (*n*=1) could eat solid food.

No signs of neurological toxicity was observed. One patient in Protocol C exhibited transient haematological toxicity upon termination of radiotherapy (leukocytes 1.8, thrombocytes 86), which was resolved after a week. No patient failed to complete the treatment due to toxicity. Disease progression or deterioration in performance were reasons for not completing the treatment scheduled.

In 40 patients (nine in Protocol A, 14 in Protocol B, and 17 in Protocol C) surgery was performed and radiotherapy and chemotherapy were administered. The other 15 patients (seven in Protocol A, three in Protocol B, and five in Protocol C) received a combination of chemotherapy and radiotherapy only. Surgery was considered microscopically radical in the four patients with a large T3 tumour (Patients 1, 23, 24, and 32), marginal in 30 patients (six in Protocol A, nine in Protocol B, and 15 in Protocol C), and not radical (visible residual tumour) in six patients (two in each protocol). The preoperative doxorubicin administration together with concomitant hyperfractionated radiotherapy thus converted generally unresectable tumours into resectable tumours.

After completion of the local therapy, 22 patients (six in Protocol A, seven in Protocol B, and nine in Protocol C) were able to continue with doxorubicin treatment (20 mg i.v. weekly) (Table 1).

### Survival

The median survival was 3.5 months (range 0–156) in Protocol A, 4.5 months in Protocol B (range 0–102), and 2 months in Protocol C (range 0–28). The numbers of patients with distant metastases at diagnosis were 6, 4, and 7, in Protocols A, B and C, respectively. Nine patients (16%) survived for over a year, evenly distributed between the three series. Only one of these nine patients died due to local failure (at 13 months; Protocol B). Two patients died due to distant metastases at 13 months and 15 months, and one patient due to subcutaneous metastases of a coexisting oxyphil carcinoma (Azkanazy cell cancer) at 21 months. All five patients (9%) who survived longer than 2 years (49 months+, 64 months, 65 months+, 102 months+, 156 months+) showed no signs of recurrence. The poor survival in Group C of only 2 out of 22 (9%) patients was disappointing with respect to the improved local control.

All these patients had received the prescribed regimen including surgery, except for Patient 2 (Protocol A), who had a large intrathyroidal tumour. All the other patients had tumours with extrathyroidal growth, for which surgery was considered marginally radical. The initial and also predominant site of metastases was always the lungs. Death resulting from intercurrent disease was attributed in two cases to cardiovascular disease (Numbers 10 and 27), and in the remaining cases to infections. No apparent relation between deaths attributed to intercurrent disease and therapy seemed to exist.

### Local tumour control

The individual local tumour response to the three regimens employed is presented in Table 1. Death due to local failure occurred in 13 (24%) of the 55 patients evaluated (in Protocol A 6 out of 10; B 2 out of 17; C 5 out of 22). Conversely, there were no signs of local remnant tumour or local recurrence in 33 (60%) of the patients distributed as follows: five out of 16 in Protocol A, 11 out of 17 in Protocol B, 17 out of 22 in Protocol C. This indicates a strong correlation between local tumour control and acceleration of radiotherapy (Fisher's exact test *P*=0.017; χ^2^ test for trend *P*=0.004). The improved local control could not be attributed to any differences in terms of local tumour extension between the three series or to differences in the number of patients operated on. Additional surgery was, however, a prerequisite for eradication of local disease.

In the 40 patients undergoing additional surgery, 33 patients (83%) showed no signs of local recurrence. The local control varied significantly between the three protocols. In Protocol A, 5 out of 9 did not show any signs of local recurrence, while the corresponding figures in Protocol B and C were 11 out of 14 and 17 out of 17, respectively (Fisher's exact test *P*=0.005; χ^2^ test for trend *P*=0.015). Although additional surgery was a prerequisite for local tumour control, the trend analysis demonstrated that improved local tumour control was correlated to more accelerated therapy, also when surgery was taken into consideration. It should be emphasised that none of the 17 patients in Protocol C had a local tumour remnant or local recurrence when surgery was feasible. On the other hand, in Protocol A two patients with pulmonary metastases at diagnosis (Patients 14 and 15) did not undergo surgery and finally succumbed due to local failure.

## DISCUSSION

The primary aim of preventing patients with ATC from dying due to suffocation caused by local tumour invasion, was achieved in Protocol C. The present multimodal treatment of ATC seems to be feasible and effective, despite the patients' high age and locally advanced disease. There was a significant positive correlation between accelerated radiotherapy and local tumour control. None of the 17 patients in Protocol C, i.e. receiving the most accelerated hyperfractionated radiotherapy, and subsequent surgery had a local remnant tumour or suffered local recurrence.

Macroscopically radical surgery is a prerequisite for local cure, since all 33 patients showing no signs of remnant or recurrent local tumour growth had had surgery. It appears that the surgery does not need to be microscopically radical as only three fulfilled this criterion; one in Protocol A, and two in Protocol B. A conceivable alternative to a preoperative radiation dose of 46 Gy would be a dose of 68–70 Gy with the same fractionation (1.6 Gy×2) but without succeeding operation. Such an alternative would probably not be feasible for this elderly patient population and furthermore the eradication of large thyroid tumours would probably be less effective than that of the present multimodal strategy (Mitchell *et al*, 1999).

No differentiated thyroid carcinomas with small anaplastic foci, which have a more favourable prognosis, were included in the present study. Although only advanced ATC were included, nine patients (16%) had a survival exceeding one year. All five patients (9%) with a survival exceeding 2 years seem to be cured.

Regardless of the poor prognosis for ATC, the possibility of saving patients from suffocation justifies the combined local treatment, also in selected patients with metastatic disease.

We have shown that weekly doxorubicin (20 mg) therapy could be performed without severe toxicity within a multimodal approach, even when the most accelerated hyperfractionated radiotherapy (1.6 Gy×2/day) was used. In a previous study, performed at the same centres, presented by Werner *et al* (1984) and reviewed by Nilsson *et al* (1998), the same regimen as that used in Protocol A (1 Gy×2/day) in the current study, apart from the chemotherapy, was employed. The chemotherapy then consisted of BCF (bleomycin, 5 mg day 1, cyclophosphamide 200 mg day 1, 5-fluorouracil, 500 mg every other day). Only nine out of 25 (36%) patients died of local failure in that study. Three patients were considered cured after a follow-up of 6 years with no recurrence. Several of the patients included suffered from severe therapy-related toxicity (epithelitis or mucosal ulcerations in the mouth and throat) which caused interruptions of the treatment. Following a report by Kim and Leeper (1983), we replaced the BCF with weekly administered doxorubicin. In addition to weekly doxorubicin, Kim and Leeper (1983; 1987) used hyperfractionated but not accelerated radiotherapy, and reported results similar to those obtained in our protocol with BCF but with less toxicity. Our aim in this present study was to prospectively evaluate the treatment and change only one modality at a time; this practice will enable assessment of the various components in the multimodality treatment regimen.

No response was observed in distant metastases in the present study, nor in another study employing doxorubicin (60 mg m^2^), cisplatin (90 mg m^2^) and local radiation (Schlumberger *et al*, 1991). The use of a more aggressive cytostatic regimen in a multimodal approach is therefore not justified, except possibly in younger subjects in good performance upon termination of local therapy. Such a strategy would not compromise the completion of the local therapy, which, however, comes into conflict with starting the systemic therapy when the metastases are still small or not yet apparent. The observed relative ineffectiveness of antineoplastic agents in ATC suggests an active role for one or more cellular mechanisms associated with chemotherapy resistance (Satake *et al*, 1997; Ain, 1998). Apart from providing an explanation of the failure of systemic chemotherapy, such mechanisms might provide appropriate targets for inactivation in order to restore clinical response to standard chemotherapies (Ain, 1998).

Although no form of chemotherapy has yet been found to result in improvement in survival or to have a substantial effect on established metastases, new chemotherapeutic agents should be tested (Ain, 1998). New modalities such as inhibitors of angiogenesis, might in the future, also prove to be useful tools for control of the growth of ATC (Hama *et al*, 1997).
